# Supplementary material for: Artificial neural networks for short-term forecasting of cases, deaths, and hospital beds occupancy in the COVID-19 pandemic at the Brazilian Amazon
Source: PLoS One. 2021 Mar 11;16(3):e0248161. doi: 10.1371/journal.pone.0248161 (PMC7951831; doi:10.1371/journal.pone.0248161)
Supplement: S1 File — (PDF) [file pone.0248161.s007.pdf]

Certificate of Translation

On behalf of the Voluntary Translation of Informative Materials related to COVID-19 Project at the Federal University of Bahia, we inform that the research article "**Artificial neural networks for short-term forecasting of cases, deaths, and hospital beds occupancy in the COVID-19 pandemic at the Brazilian Amazon"** was translated into English by our team of training translators. We also inform that, as it is a pedagogic project, the text was closely supervised and revised by the professors responsible for the project.

November 27 2020
Salvador - Bahia - Brazil
Universidade Federal da Bahia

Prof. Dr. Monique Pfau

Prof. Dr. Lucielen Porfirio

Prof. Me. Daniel Vasconcelos B. Oliveira

Prof. Dr. Feibriss Henrique Meneghelli Cassilhas
