# Supplementary material for: Artificial neural networks for short-term forecasting of cases, deaths, and hospital beds occupancy in the COVID-19 pandemic at the Brazilian Amazon
Source: PLoS One. 2021 Mar 11;16(3):e0248161. doi: 10.1371/journal.pone.0248161 (PMC7951831; doi:10.1371/journal.pone.0248161)
Supplement: Attachment — Submitted filename: Response to Reviewers.docx [file pone.0248161.s008.docx]

**Response to Reviewers**

***Journal Requirements:***

1. Please ensure that your manuscript meets PLOS ONE's style requirements, including those for file naming. The PLOS ONE style templates can be found at

https://journals.plos.org/plosone/s/file?id=wjVg/PLOSOne_formatting_sample_main_body.pdf

and

https://journals.plos.org/plosone/s/file?id=ba62/PLOSOne_formatting_sample_title_authors_affiliations.pdf

***RESPONSE: We have reviewed and edited the manuscript according to the journal's recommendations.***

2. We suggest you thoroughly copyedit your manuscript for language usage, spelling, and grammar. If you do not know anyone who can help you do this, you may wish to consider employing a professional scientific editing service.

***RESPONSE: According to reviewers the manuscript was presented in an intelligible fashion and written in standard English. We have submitted the manuscript to an official translation service at the Federal University of Bahia, Brazil. We will send the translation declaration as an attached file.***

3. We note that Figure 1 in your submission contain map images which may be copyrighted. All PLOS content is published under the Creative Commons Attribution License (CC BY 4.0), which means that the manuscript, images, and Supporting Information files will be freely available online, and any third party is permitted to access, download, copy, distribute, and use these materials in any way, even commercially, with proper attribution. For these reasons, we cannot publish previously copyrighted maps or satellite images created using proprietary data, such as Google software (Google Maps, Street View, and Earth). For more information, see our copyright guidelines: http://journals.plos.org/plosone/s/licenses-and-copyright. We require you to either (1) present written permission from the copyright holder to publish these figures specifically under the CC BY 4.0 license, or (2) remove the figures from your submission:

***RESPONSE: Thank you for the careful revision, but the Figure 1 was created by the manuscript's authors and they own its use rights.***

***Review Comments to the Author - Reviewer #1:***

1 - More illustration on the strength of ANNs are expected in the Introduction section

***RESPONSE: Thank you for the suggestion. We have added in the introduction section the following text (line 70), reinforcing the strength of the ANNs:***

***“The main characteristic of ANN is self-learning without prior knowledge of the complex non-linear relationships that exist between the input and output variables [22]. This is due to the massive and parallel processing of neurons and the tolerance to noise [23]. In addition, this technique captures small distortions in the observed data and transfers them for projections differently from mechanistic models [24]. Another advantage is that this type of approach also makes it possible to use several predictor variables simultaneously, such as demographic data and incidence curves, which helps in capturing the dynamics of virus transmission in the cities over time [25,26].”***

2 - Additionally, more references on the application of ANNs and the used criteria for measuring goodness-of-fit should be acknowledged, such as:

Modeling nonlinear relationship between crash frequency by severity and contributing factors by neural networks. Analytic Methods in Accident Research, 2016, 10: 12-25.

Macro and micro models for zonal crash prediction with application in hot zones identification. Journal of Transport Geography, 2016, 54: 248-256.

Bayesian spatial-temporal model for the main and interaction effects of roadway and weather characteristics on freeway crash incidence. Accident Analysis and Prevention, 2019, 132, 105249.

***RESPONSE: Thank you for the suggestion. The suggested references were added in the materials and methods section, line 212:***

***“For each application and data set observed, it is necessary to choose the most appropriate technique among the many available. In this case, different performance metrics can be used as selection criteria. Zeng and Wen used Mean Absolute Deviance (MAD) and Mean Squared Prediction Error (MSPE) [25,46], and, in an epidemiological context, Chowell used the Root Mean Squared Error (RMSE), Mean Squared Error (MAE), and Mean Absolut Percent Error (MAPE) [15].”***

3 - In the ANN modeling section, a figure showing how the output and inputs connected would present the model more explicitly.

***RESPONSE: We have added the figure (Fig. 3) as suggested by the reviewer in the ANN modeling subsection.***

4 - The Broyden-Fletcher-Goldfarb-Shanno iterative algorithm used for ANN training is rare in the previous studies. A detailed description of this algorithm is anticipated.

***RESPONSE: We have added a brief explanation of the BFGS algorithm and quote a reference that shows the detailed operation of the algorithm in the ANN modeling subsection, at line 178, as follow:***

***“The BFGS memoryless quasi-Newton was successfully used for minimizing errors on artificial neural networks. The Quasi-Newton method is a method that is used when the calculation of the Hessian matrix is difficult or time-consuming. This method has a rapid convergence when compared with the method of gradient descent [44].”***

5 - It would be interesting and further demonstrate the advantages of ANNs to compare their performance with that of time series models that are used in the previous studies on forecasting COVID-19 cases.

***RESPONSE:***

***Thank you for the suggestion. Unfortunately, we did not found studies with similar methodology and proposal to compare. But we have added the information regards the work of Petropoulos F & Makridakis S. [54] that performs an analysis of different scenarios but with different day intervals and without goodness-of-fit analysis, which prevents a proper comparison.***
